# Neuroprotective Effect of Subdural Infusion of Serp-1 in Spinal Cord Trauma

**DOI:** 10.3390/biomedicines8100372

**Published:** 2020-09-23

**Authors:** Jacek M. Kwiecien, Wojciech Dabrowski, Bryce J. Kwiecien-Delaney, Christian J. Kwiecien-Delaney, Dorota Siwicka-Gieroba, Jordan R. Yaron, Liqiang Zhang, Kathleen H. Delaney, Alexandra R. Lucas

**Affiliations:** 1 Department of Pathology and Molecular Medicine, McMaster University, Hamilton, ON L8S 4K1, Canada; delaneyk@mcmaster.ca; 2 Department of Anaesthesiology and Intensive Therapy, Medical University of Lublin, 20-090 Lublin, Poland; w.dabrowski5@gmail.com (W.D.); dsiw@wp.pl (D.S.-G.); 3 Faculty of Science, McMaster University, Hamilton, ON L8S 4K1, Canada; brykwiec@umich.edu (B.J.K.-D.); ckdftw@gmail.com (C.J.K.-D.); 4 Center for Personalized Diagnostics and Center for Immunotherapy, Vaccines and Virotherapy, Biodesign Institute, Arizona State University, Tempe, AZ 85287, USA; jyaron@asu.edu (J.R.Y.); liqiang.zhang@asu.edu (L.Z.); arlucas5@asu.edu (A.R.L.)

**Keywords:** spinal cord injury, macrophage, neuroprotective therapy, Serp-1, cavity of injury, histology

## Abstract

Spinal cord injury (SCI) initiates a severe, destructive inflammation with pro-inflammatory, CD68+/CD163−, phagocytic macrophages infiltrating the area of necrosis and hemorrhage by day 3 and persisting for the next 16 weeks. Inhibition of macrophage infiltration of the site of necrosis that is converted into a cavity of injury (COI) during the first week post-SCI, should limit inflammatory damage, shorten its duration and result in neuroprotection. By sustained subdural infusion we administered Serp-1, a Myxoma virus-derived immunomodulatory protein previously shown to improve neurologic deficits and inhibit macrophage infiltration in the COI in rats with the balloon crush SCI. Firstly, in a 7 day long study, we determined that the optimal dose for macrophage inhibition was 0.2 mg/week. Then, we demonstrated that a continuous subdural infusion of Serp-1 for 8 weeks resulted in consistently accelerated lowering of pro-inflammatory macrophages in the COI and in their almost complete elimination similar to that previously observed at 16 weeks in untreated SCI rats. The macrophage count in the COI is a quantitative test directly related to the severity of destructive inflammation initiated by the SCI. This test has consistently demonstrated anti-inflammatory effect of Serp-1 interpreted as neuroprotection, the first and necessary step in a therapeutic strategy in neurotrauma.

## 1. Introduction

In a recent systematic study we demonstrated that the SCI initiates by day 3, an Inflammatory Phase with severe, phagocytic, CD68+/CD163−, macrophage infiltration of the site of injury that becomes converted into a cavity of injury (COI) [1,2,3,4]. Macrophage infiltration in the COI occurs with initially marked elevation of pro-inflammatory cytokines, chemokines and other factors. Although the numbers of macrophages and levels of pro-inflammatory factors decline after 4 weeks post-SCI, presumably influenced by a spinal cord anti-inflammatory reaction, specifically astrogliosis [4], damage to the spinal cord around the COI and severe inflammation therein is likely considerable [5,6] and an anti-inflammatory treatment would be neuroprotective.

Infiltration by phagocytic macrophages in the COI is an obvious and characteristic feature of post-SCI pathology and their count can be performed in a standardized fashion in luxol fast blue and hematoxylin and eosin (LFB + H&E) stained cross sections of the injured spinal cord. The macrophage count in the COI can serve as a quantitative test directly addressing the severity of destructive inflammation post-SCI [3,4,7]. Therefore, a reduction in counts of macrophages in the COI of rats administered an anti-inflammatory treatment would be considered neuroprotective [4].

Previous studies on anti-inflammatory drugs demonstrated that subdural infusion in the vicinity of the crush lesion, allows for lowering of numbers of macrophages in the COI presumably by simple diffusion of administered dexamethasone, M-T7 or Serp-1 into the fluid filled COI [1,2,3]. We recently reported that administration of the Myxoma virus-derived immune modulating serpin, Serp-1 [8], infused locally for 7 days starting immediately after balloon crush SCI, reduced inflammation and improved neurologic scores in rat models [3]. In another experiment, Serp-1 delivered in the chitosan hydrogel in the acute lesion created by the dorsal spinal column crush also resulted in anti-inflammatory effect associated with reduction of the size of the lesion and improvement in clinical scores [9]. Serp-1 binds and inhibits tissue- and urokinase-type plasminogen activators, plasmin in the thrombolytic cascade, and thrombin and Factor X in the coagulation/thrombotic cascade [10]. This serpin protein biologic has been shown to reduce vasculitis in vascular injury and in organ transplant models [11]. Serp-1 was also tested in a Phase 2 clinical trial in patients with unstable coronary syndromes and coronary stent implant where it significantly reduced markers of heart damage [12]. Serp-1 is thus a promising biologic for use in a variety of inflammatory conditions, including SCI [3,9].

A successful preliminary pre-clinical study mandated by the Food and Drug Administration [13] of an anti-inflammatory/neuroprotective compound needs to demonstrate its effectiveness, the optimal route of administration, the optimal effective dose and the duration of treatment necessary to eliminate destructive inflammation initiated by neurotrauma [4]. Previously, we have established a novel test, phagocytic macrophage count in the cavity of injury (COI) [3,4] which is directly relevant to the severity of destructive inflammation initiated by the SCI and allowed us to determine anti-inflammatory effectiveness of Serp-1, and its optimal route of administration defined as subdural infusion [3]. We have also determined that a successful neuroprotective treatment of destructive inflammation post-SCI, will need to be considerably longer than 1–2 weeks [1,2,3] to eliminate inflammation. This is related to the slowing of phagocytosis of myelin-rich necrotic debris and red blood cells in treated rats [1,2,3]. We postulate that elimination of the destructive inflammation from the COI will render this cavity amenable to implantation of a functional bridge for axonal regeneration across this fluid-filled cavity in similar fashion to that achieved in the Long Evans Shaker (LES) dysmyelinated rat implanted with the choroid plexus [14].

In this study, using the macrophage count test, we determined the optimal dose of Serp-1 administered in subdural infusion and the duration of the infusion to accelerate elimination of macrophage infiltration in the COI thus resulting in neuroprotection.

## 2. Experimental Section

This study was performed in 2 phases to determine; (1) the dose effect of Serp-1 infused subdurally for 1 week to inhibit macrophage infiltration of the cavity of injury (COI) post-SCI, and (2) the duration of such treatment by the optimal dose of Serp-1 leading to elimination of macrophages from the COI.

### 2.1. Ethical Considerations

Experiments using male, 16 weeks old Long Evans rats, 370–410 g, were approved by the Animal Research Ethics Board at McMaster University according to the Guides and Regulations of the Canadian Council of Animal Care. Given the invasive nature of the SCI, detection of the following End Point; lethargy, marked dehydration, hypothermia, and/or ruptured urinary bladder was followed by humane euthanasia and the rat was excluded from the study (*n* = 8).

### 2.2. Serp-1 Protein Expression and Purification

Recombinant Serp-1 (m008.1L; NCBI Gene ID# 932146) was expressed and secreted by a Chinese hamster ovary (CHO) cell line (Viron Therapeutics Inc., London, ON, Canada). GMP-compliant purification was performed with greater than 95% purity as determined by Coomassie-stained SDS PAGE and reverse-phase HPLC. Serp-1 was endotoxin-free by LAL assay and stocked at −80 °C in 100 mM citrate buffer (pH 6.5) at a concentration of 1.8 mg/mL [12].

### 2.3. Balloon Crush SCI in the Rat

The surgical procedure involved the balloon crush SCI and subdural infusion was performed in 58 rats (Table 1) and has been previously described [1,2,3,4,15].

Briefly, 58 rats were induced for SCI surgery in 5% isoflurane in 95% oxygen and maintained under 4% isoflurane in 96% oxygen. The laminectomy was created at T10 vertebrae and a 3Fogarty catheter (Balton, Warsaw, Poland) inserted towards the head over the dura to position the balloon over the mid-thoracic spinal cord. The balloon was inflated with 20 µL saline for 5 s, deflated and removed. For subdural infusion, a small cut was created in the dura over the dorsal spinal cord in the laminectomy and a rat intrathecal catheter (Alzet^®^, Durect Corporation, Cupertino, CA, USA) inserted over the spinal cord cranially to approximate the catheter tip with the site of the crush injury. The other end of the catheter was connected to the osmotic pump (Alzet) placed under the skin of the flank. For determination of the optimal dose of Serp-1, 0.008 mg, 0.04 mg or 0.2 mg of the protein in saline were loaded into 2ML1 osmotic pumps providing a constant flow of 10 µL/h over 7 days (Alzet). For determination of the duration of infusion required to eliminate macrophage infiltration from the cavity of injury (COI), 2ML4 pumps were loaded with 0.8 mg Serp-1 or saline providing a constant flow of 2.5 µL/h over 28 days. At 28 days post-SCI, the spent pumps were replaced by newly loaded ones via a skin incision and under isoflurane anesthesia. During the total 56 days treatment, 1.6 mg of Serp-1 was infused. Prior to awakening from anesthesia, all rats received injection of 0.4 mL ketoprofen analgesic (10 mg/mL, Anafen, Merial Canada, Inc., Baie d’Urfe, QC, Canada) and 5 mL saline subcutaneously.

### 2.4. Post-Operative Care and Clinical Testing

Post-surgical rats were attended 1–2 times per day. Hydration status, body temperature and presence of lethargy were assessed. Rats with dilated urinary bladder were gently voided and rats with hemorrhagic urine treated by intramuscular injection of 50 µL enrofloxacin (50 mg/mL, Baytril^®^, Mississauga, ON, Canada) for 3–5 days until blood cleared from urine. The function of the urinary bladder typically returned during the second week post-SCI.

To detect therapeutic effect of Serp-1 in SCI rats a simplified hind end locomotor test with 7 scoring levels was developed previously [7,9] and used every day starting with day 1 post-SCI to assess the locomotor function of the hind legs as described in the Table 2.

In addition, a pinch withdrawal reflex test (Table 3) previously developed [7,9] was used every day in post-SCI rats.

### 2.5. Pathology

At 7 days post-SCI in the Serp-1 dosing study and at 14, 28, and 56 days post-SCI in the effective duration of administration study, rats were overdosed with 80 mg/kg body weight sodium pentobarbital, intraperitoneal, and the whole body perfusion with saline followed by formalin [4]. The spines were dissected, post-fixed in formalin for 1–2 days and decalcified in formalin supplemented with 8% ethylenediaminetetraacetic acid (EDTA, Bioland Scientific, Paramount, CA, USA) [3,4]. When soft, the spines were cut perpendicularly at 3 mm thick, consecutive segments including the laminectomy and the site of injury. Eight consecutive segments were processed, embedded in paraffin wax, 5 µm thick sections mounted on glass slides and stained with luxol fast blue and counterstained with hematoxylin and eosin (LFB + H&E). Additional sections were labelled with a primary anti-CD68 antibody and brown color developed as described previously [3].

#### Macrophage Counts in the Cavity of Injury

The LFB + H&E stained sections of the spinal cord were analyzed under a light 50i Eclipse Nikon microscope by an experienced experimental neuropathologist and one area with the cavity of injury (COI) photographed per section with 40× objective; 3–5 sections per rat as described previously [3,4]. The images measured 225 × 300 µm and included the periphery of the COI with adjacent spinal cord tissue taking 20% of the area of the image. Macrophages; large cells with a large oval nucleus and abundant vacuolated cytoplasm with blue granules of myelin and/or with red blood cells, were counted and the average for each rat in a treatment group (Table 1) was averaged and standard deviation calculated [3,4].

### 2.6. Statistical Analysis

The statistical analysis was performed using STATISTICA software, version 13.0 (StatSoft, USA) and GraphPad Prizm v8.4.3 with the significance level of 0.05. Normal distribution was assessed using Shapiro-Wilk and Kolmogorov-Smirnov tests. Variables were presented as the mean and stdev due to normal distribution and differences were tested using Student parametric *t*-test and one-way and two-way ANOVA with Holm-Sidak and Sidak post-hoc tests, respectively, where indicated.

## 3. Results

**Figure 1:**
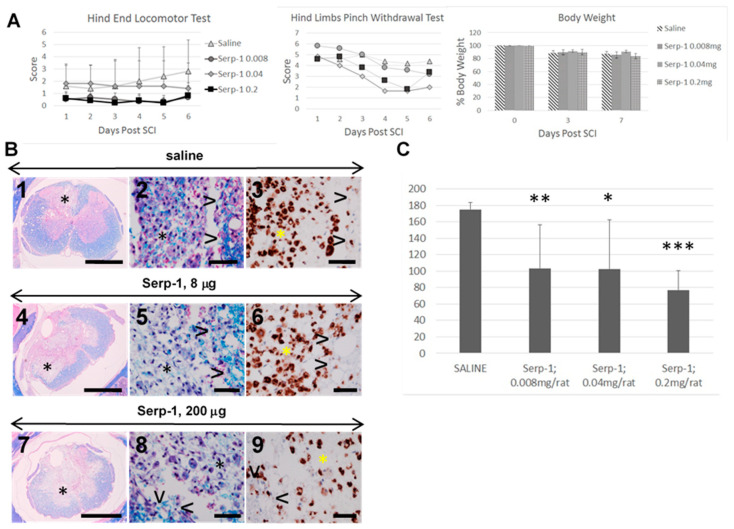
Effect of rising dose of Serp-1 on macrophage infiltration at 7 days post-injury. (**A**) Clinical scores. Clinical testing on SCI rats was performed starting at day one post-SCI and then each day for 6 days. The hind end locomotor test was performed as described in Table 2 and the hind limbs pinch withdrawal test was performed as described in Table 3. The daily scoring results in graphs indicate no beneficial effect of Serp-1 treatment. The body weights were taken before the SCI surgery then at days 3 and 7 post-SCI and are expressed as percent of the pre-SCI body weight of individual rats. (**B**) Histologic analysis. Histology of spinal cords 7 days post-SCI infused subdurally with saline (1–3), 8 µg Serp-1 (4–6) and 200 µg Serp-1 (7–9) reveals large areas of damage including cavities of injury (COI, *) delineated by surrounding tissue (>) and infiltrated by numerous macrophages laden with blue granules of myelin in luxol fast blue and hematoxylin and eosin (LFB + H&E stain) (middle column). Large proportion of macrophages in the COI are CD68+ (right column, brown color). While in the saline treatment macrophages in the COI are numerous (2,3), in the Serp-1 treatment at 200 µg the numbers of macrophages are lower on CD68+ labelling (9) and the amount of un-phagocytized myelin-rich necrotic debris is markedly greater (8). Luxol fast blue with hematoxylin and eosin counterstain (LFB + H&E)—two left columns. Anti-CD68 antibody stain, the right column. Size bars-1000 µm the left column, 50 µm two right columns. (**C**) Macrophage count in the COI. The macrophage counts in the COI of SCI rats infused for 7 days are averaged and stdev calculated for saline treatment and for Serp-1 treatment totaling 0.008, 0.04 and 0.2 mg. The statistical differences for the effect of each treatment vs. saline are; * for *p* < 0.05, ** for *p* < 0.01 and *** for *p* < 0.001.

The results of the 7 day long dosing study are summarized in the Figure 1.

### 3.1. Clinical Testing, 7 Days Study

The hind end locomotor test revealed no therapeutic effect in rats treated with 0.008–0.2 mg Serp-1 sustained subdural infusion. While the scores for saline infused rats improved, the scores for Serp-1 infused rats remained flat. This pattern of changes in the hind end locomotor scores is not supported by the progression in pathogenesis of SCI during the first 7 days of its duration when the severity of pro-inflammatory, CD68+/CD163− macrophage infiltration is rapidly developing and indicates a marked worsening of inflammatory disease initiated by neurotrauma [4]. The hind limb pinch withdrawal test revealed the decline in scores for all treatment groups and no apparent therapeutic effect for Serp-1 treatments. All rats lost body weight after the surgery, reaching approximately 90% at 3 days post-SCI and approximately 85% at day 7. The initiation of the subdural infusion in a similar fashion indicated a negative effect of SCI surgery but no additional effect of Serp-1 treatment.

### 3.2. Histologic Analysis, 7 Days Study

Inflammatory infiltration in form of the COI, (asterix in Figure 1B) and of arachnoiditis obliterated large areas of the spinal cord 7 days post-SCI. In the COI of rats infused with saline there were numerous large phagocytic cells with oval, sometimes subcleaved nucleus and abundant cytoplasm containing blue granules of myelin and/or red blood cells (Figure 1B2). These cells were tightly packed in the periphery of COI leaving little unphagocytized myelin-rich material and scattered blood cells. Large proportion of infiltrating cells were CD68+ (Figure 1B3), marker for pro-inflammatory macrophages [16,17]. The numbers of phagocytic macrophages were lower in the COI of rats infused with 8 µg of Serp-1 (Figure 1B5,6) and markedly lower in rats infused with 200 µg of Serp-1 (Figure 1B8,9) with large amounts of myelin-rich necrotic debris and red blood cells between scattered macrophages (Figure 1B8) further supporting the observation of inhibition of active macrophage phagocytosis.

### 3.3. Macrophage Counts, 7 Days Study

**Figure 2:**
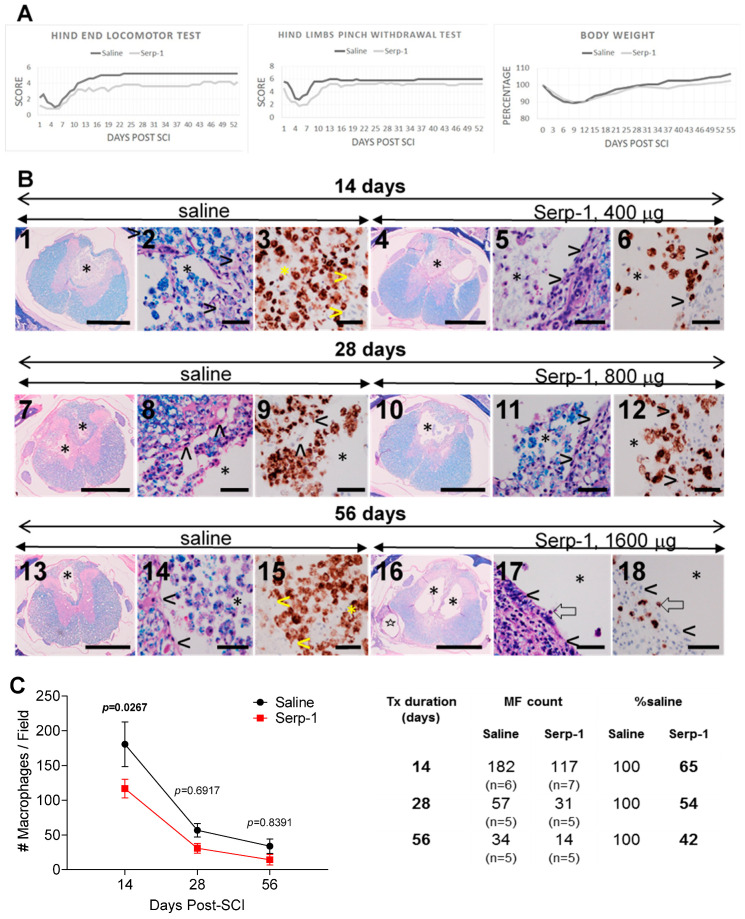
Effect of Serp-1 in 56 days treatment. (**A**) Clinical scores. The clinical scores in the hind end locomotor test and in the hind limb pinch withdrawal test for Serp-1 treatment were performed daily during the 56 day study. At 28 days post-SCI the spent 2ML4 pump was replaced with a fresh 2ML4 pump with the same amount of Serp-1 or saline and maintained for additional 28 days, total 56 days infused with a total of 1.6 mg Serp-1. There is no beneficial effect of Serp-1 infusion. The stabilization of scores during the 4th week for the hind end locomotor test and during the 3rd week for the hind limb pinch withdrawal test does not reflect the severe pathology observed histologically during the remaining 4–5 weeks of the study. The body weights, taken every 3rd day are expressed as % of pre-surgical body weight for each rat and averaged. (**B**) Histologic analysis. The low magnification micrographs for the saline (1,7,13) and for Serp-1 (4,10,16) infusion show cavities of injury (COI, *) delineated by spinal cord (>) in high magnification micrographs. The COI contain multiple large phagocytic cells with internalized blue granules of myelin and/or red blood cells. A large proportion of cells in the COI are positive for CD68+ antibody (brown color) and are consistently more numerous in the saline treatment than in the Serp-1 treatment. Myelin-rich necrotic debris are not evident between phagocytic cells in saline-treated rats but abundant at 14 and 28 days although not at 56 days post-SCI in Serp-1 treated rats. Open arrow (⇦) in 17 and 18 indicate single phagocytic, CD68+ macrophage. A structure indicated by star ✫ in 16) is the cross section of the intrathecal catheter placed subdurally. Size bars, 1,4,7,10,13,16; 50–1000 µm; remaining microphotographs—50 µm. Staining, 3,6,9,12,15,18-anti-CD68 antibody for pro-inflammatory macrophages; luxol fast blue with hematoxylin and eosin counterstain (LFB + H&E)—remaining microphotographs. (**C**) Macrophage counts in the cavity of injury. The macrophage counts in the cavity of injury (COI) were performed for Serp-1 and for saline treatments at 14, 28 and 56 days post-SCI. Although the statistical differences are not obvious at these time points, there is the reduction of the numbers of macrophage counts to 65%, 54% and 42% in Serp-1 treatment compared to saline treatment.

The standardized macrophage counts were lower for all levels of treatment with Serp-1 (Figure 1C) with the greatest inhibitory effect at 200 µg/week (*p* < 0.001) and this amount of Serp-1 was used in the next phase of the study to determine the therapeutic effect of the prolonged infusion (See Figure 2). It was shown previously that subdural infusion of 1.0 mg/week of Serp-1 did not result in greater inhibition of macrophage infiltration in the COI [3].

The results of the 56 day long duration of administration study are summarized in Figure 2.

### 3.4. Clinical Testing, 56 Days Study

The scores of the hind end locomotor test (Figure 2A) were negatively affected by the balloon crush SCI surgery and still declined during the first week in both treatments. Over the two following weeks however, the scores in both treatment groups improved and achieved a plateau during the 4th week post-SCI. The scores for the remaining 4 weeks were similar, constant and consistently higher for the saline treatment than scores for the Serp-1 treatment.

The scores from the hind limb pinch withdrawal test were summarized for both legs (Figure 2A) and revealed reduction during the first week post-SCI but then recovered during the second week and remained constant for the remainder of the study in both treatment groups. The scores for saline treatment were moderately higher than the scores for Serp-1 treatment.

The body weights in all rats consistently declined within the first 10 days post-SCI to approximately 90% of the pre-surgical body weight. During the second week however, the body weights begun to recover in a continuous fashion and achieved approximately 105% in saline treatment group and approximately 100% in Serp-1 treatment group.

### 3.5. Histologic Analysis, 56 Days Study

At 14 days post-SCI, the COIs were infiltrated with numerous phagocytic macrophages with no extracellular necrotic debris or red blood cells in saline treated rats (Figure 2B2) but lower numbers of macrophages were scattered among necrotic debris in Serp-1 treated rats (Figure 2B5) indicating inhibitory effect on macrophage infiltration and phagocytosis. At 28 days post-SCI, the numbers of macrophages were markedly lower in the COI in both treatment groups (Figure 2B7,10) and extracellular debris still evident in Serp-1 treatment (Figure 2B11). At 56 days, numbers of phagocytic macrophages were again reduced in saline treatment rats (Figure 2B13,14) and rare in Serp-1 rats (Figure 2B17,18). The COIs in the Serp-1 rats appeared as similar cavities in untreated rats at 16 weeks post-SCI [4], and were reminiscent of mature syrinxes. A large proportion of cells in the COI were CD68+ indicating their pro-inflammatory identity. The CD68+ cells were more numerous at 28 days post-SCI in the spinal cord adjacent to the COI than at 14 and at 56 (Figure 2B) days but they were not counted.

### 3.6. Macrophage Counts, 56 Days Study

The average counts of macrophages in the COI of saline infused rats declined over the period of 56 days which is consistent with the effect of the spinal cord tissue reaction, demonstrated in the previous study [4]. The counts of macrophages at 14, 28 and 56 days post-SCI were lower in Serp-1 infused rats (Figure 2C) although the statistical difference of the therapeutic effect of Serp-1 infusion was not demonstrated. However, the counts of macrophages in Serp-1 treated rats were consistently at approximately half of the counts in saline treated rats; 65% at 14 days post-SCI, 54% at 28 days and 42% at 56 days (Figure 2C) supporting the notion of the anti-inflammatory effect of Serp-1 infusion.

## 4. Discussion

A recently completed detailed study on the pathogenesis of SCI revealed that when trauma resulting in local massive injury to the white matter initiates, by day 3 post-SCI, an Inflammatory Phase characterized by severe infiltration by phagocytic CD68+/CD163− macrophages and its destructive, extraordinarily prolonged the course lasting beyond 16 weeks [4]. The destructive inflammation initiated by SCI presents an obvious target for anti-inflammatory treatments to achieve neuroprotection.

Anti-inflammatory treatments previously attempted have not benefited from sufficient understanding of the pathogenesis of SCI and high-dose, short-term intravenous infusion of methylprednisolone [18,19,20,21] has been shown to cause severe side effects [3,20,22,23,24] and has recently been discouraged [25]. Other compounds including riluzole, glibenclamide and cethrin [26], and also fumaric acid esters [27], estrogen [28], endaravone [29], mitramycine A [30], and *N*-Palmytiolethalonamine-oxazoline [31] have recently been studied in SCI animal models and clinical trials but only in short term treatments in initial stages of SCI. The effects of these experimental treatments have not been measured in a fashion addressing pathogenesis of SCI leaving a possibility that some of these compounds may potentially be found neuroprotective when tested in properly designed pre-clinical studies.

Recently elucidated pathogenesis of SCI and our previous studies on short term treatment, 1–2 weeks [1,2,3], indicate that reduction of numbers of macrophages in the COI results in slower phagocytosis of myelin-rich necrotic debris and red blood cells requiring extended treatment to enable fewer macrophages to phagocytize and remove this immunogenic material. We hypothesized that continuous infusion of Serp-1, an immunomodulating protein with anti-inflammatory action would shorten the macrophage-rich inflammation down from 16 weeks in the rat model [4] resulting in neuroprotective effect.

Despite their brevity, experiments with infusion for 7 days however, have had their use in determination for Serp-1 of the; (1) robust anti-inflammatory effect, (2) optimal route of administration as subdural infusion, demonstrated in the previous study [3] and, (3) dose effect with 0.2 mg/week showing the strongest macrophage inhibition as determined in this study. The neuroprotective effect of an anti-inflammatory treatment however, requires sufficiently long continuity of administration, at least 8 weeks, to eliminate inflammation. The subdural infusion of Serp-1 for 56 days almost completely extinguished the inflammation by lowering the numbers of macrophages in the COI to very few, similar to numbers counted at 16 weeks in untreated rats [4]. Therefore, we consider that the treatment resulted in overall inhibition and shortening of the destructive inflammation initiated by SCI and can be considered neuroprotective.

The phagocytic, pro-inflammatory macrophage count is of direct consequence to the severity of destructive inflammation initiated by SCI [4], therefore it can serve as a reliable test for anti-inflammatory and neuroprotective effect of candidate drugs in pre-clinical studies. Importantly, macrophage count-lowering effect of Serp-1 infusion was associated with persistence of myelin-rich necrotic debris among scattered macrophages at 2 and 4 at weeks post-SCI, not observed in saline treatments at these time points in this and in the previous study [4]. However, since it is based on histologic analysis of the spinal cord, the macrophage count is not suitable for clinical studies and in vivo laboratory assays measuring biomarkers of the spinal cord damage and of the severity of inflammation in the body fluids such as blood plasma and the cerebrospinal fluid need to be considered and developed for systematic evaluation of neuroprotective efficacy of candidate drugs in individual SCI patients along the duration of this very destructive and protracted inflammatory disease.

The hind end locomotor test and hind limb pinch withdrawal test have been used previously in a 7 day study where Serp-1 was infused subdurally [3] and also in a 28 day study where Serp-1 was delivered from a chitosan hydrogel injected into the dorsal column crush [9]. In both studies, a beneficial effect of Serp-1 administration was observed and supported by lower counts of macrophages in the COI infused with Serp-1 [3] and reduced size of the crush injury with Serp-1 hydrogel [9]. In the present study however, both tests revealed no higher scores for Serp-1 treatments for 7 days (Figure 1A) and for 56 days (Figure 2A) despite the macrophage counts and histologic analysis consistently indicating anti-inflammatory effect of Serp-1 infusion. It needs to be highlighted that for both Serp-1 and saline treatments, the scores in both neurological tests used in this study stabilized during the week 4 of the treatment and did not change during the remaining 4 weeks (Figure 2A) despite the histologic evidence of active inflammatory disease. Given the above considerations the interpretation of the results of two neurologic tests used remains difficult [7].

Although the experimental subjects, the SCI rats, were not randomized in this study as recommended previously [32] all rats were male LE of the same age raised in the same colony, therefore of the same quality [15]. In rats coded to obscure the identity to the examiner, the results of two simplified neurological exams and also of the macrophage count in the COI test were reliable and consistently reproducible.

## 5. Conclusions

In conjunction with the previous study [3], this preliminary pre-clinical study determined that for neuroprotective therapy the SCI in the rat model, Serp-1 needs to be delivered subdurally in vicinity of the injury in constant infusion of 0.2 mg/week per rat for at least 8 weeks.

## Figures and Tables

**Table 1:** Rats with spinal cord injury infused subdurally with Serp-1.

Treatment	Duration Days	# Rats	Osmotic Pump	Total Serp-1 (mg)
Saline	7	5	2ML1	0
Serp-1, 0.008 mg	7	6	2ML1	0.008
Serp-1, 0.04 mg	7	5	2ML1	0.04
Serp-1, 0.2 mg	7	6	2ML1	0.2
Saline	14	6	2ML4	0
Serp-1, 0.2 mg/week	14	7	2ML4	0.4
Saline	28	7	2ML4	0
Serp-1, 0.2 mg/week	28	6	2ML4	0.8
Saline	56	5	2ML4 × 2	0
Serp-1, 0.2 mg/week	56	5	2ML4 × 2	1.6

**Table 2:** Scoring of neurological deficits in the hind end locomotor test (HE test) in spinal cord injury rats.

Score	Description
0	Both hind legs have no motion, extended backwards.
1	One hind leg has flexing motion caudal to the level of the hip joint, with the plantar surface of the foot up, no weight support.
2	Both legs have flexing motion caudal to the hip, with the plantar surface of the foot up, no weight support **or** one leg has flexing motion beyond the hip, nobody support, the other leg no motion.
3	One leg has flexing motion beyond the hip, with the dorsal surface of the foot up, no weight support, the other leg has flexing motion caudal to the hip, with the plantar surface of the foot up; **or** one leg has flexing motion beyond the hip, with dorsal surface of the foot up, with body weight support but the other leg has no motion.
4	Both legs have flexing motion beyond the hip, with the dorsal surface of the foot up, but no body weight support; **or** one leg with the flexing motion beyond the hip with body support and the other leg with flexing motion caudal to the hip, with the plantar surface of the foot up, but no body weight support.
5	One leg has flexing motion beyond the hip with body weight support, the other leg flexing motion beyond the hip, with the dorsal surface of the foot up, but no body support.
6	Normal gait, no apparent weakness or proprioceptive deficits.

**Table 3:** Scoring of the toe pinch withdrawal test in spinal cord injury (SCI) rats.

Score	Description
0	No toe retraction.
1	Weak retraction, no jerking.
2	Weak retraction with jerking.
3	Strong/normal retraction with jerking.
